# The Relationship between Sleep Parameters Measured by Polysomnography and Selected Neurotrophic Factors

**DOI:** 10.3390/jcm13030893

**Published:** 2024-02-03

**Authors:** Marcin Sochal, Agata Binienda, Aleksandra Tarasiuk, Agata Gabryelska, Piotr Białasiewicz, Marta Ditmer, Szymon Turkiewicz, Filip Franciszek Karuga, Jakub Fichna, Adam Wysokiński

**Affiliations:** 1 Department of Sleep Medicine and Metabolic Disorders, Medical University of Lodz, 90-419 Lodz, Poland; agata.gabryelska@gmail.com (A.G.); piotr.bialasiewicz@umed.lodz.pl (P.B.); turkiewiczszymon99@gmail.com (S.T.); filip.karuga@stud.umed.lodz.pl (F.F.K.); 2 Department of Biochemistry, Medical University of Lodz, 92-215 Lodz, Poland; agata.binienda@gmail.com (A.B.); tarasiuk.aleksandra@gmail.com (A.T.); jakub.fichna@umed.lodz.pl (J.F.); 3 Department of Old Age Psychiatry and Psychotic Disorders, Medical University of Lodz, 92-216 Lodz, Poland; adam.wysokinski@umed.lodz.pl

**Keywords:** BDNF, sleep, neurotrophins

## Abstract

Background: The molecular underpinnings of insufficient sleep remain underexplored, with disruptions in the neurotrophic signaling pathway emerging as a potential explanation. Neurotrophins (NTs), including brain-derived neurotrophic factor (BDNF), neurotrophin-3 (NT3), neurotrophin 4 (NT4), and glial-cell-line-derived growth factor (GDNF), play crucial roles in nerve cell growth and repair. However, their associations with sleep patterns are poorly understood. This study aimed to investigate the relationship between the chosen neurotrophins and objective sleep parameters. Methods: The study involved 81 participants subjected to polysomnography (PSG). Blood samples were collected after PSG. The mRNA expression and serum protein concentrations of BDNF, GDNF, NT3, and NT4 were measured using real-time quantitative reverse-transcription PCR (qRT-PCR) or enzyme-linked immunosorbent assay (ELISA) methods, respectively. Results: BDNF and NT3 proteins were negatively correlated with NREM events, while NT4 protein positively correlated with REM events. Electroencephalography power analysis revealed BDNF protein’s negative correlation with delta waves during rapid eye movement and non-rapid eye movement sleep. Conclusion: The study highlights associations between neurotrophins and sleep, emphasizing BDNF’s role in regulating NREM and REM sleep. The EEG power analysis implicated BDNF in delta wave modulation, shedding light on potential neurotrophic mechanisms underlying sleep effects on cognitive and mood processes.

## 1. Introduction

Neurogenesis occurs throughout human life and plays an important role in the hippocampus, which is responsible for learning and memory. One factor that affects it is sleep. Sleep disorders are a common public health problem, experienced by almost one third of the population [1]. The most common cause is insufficient sleep time, which often accompanies mental disorders, particularly depressive and anxiety disorders [2]. Despite intensive research, the exact mechanisms by which sleep disorders can lead to mental disorders, especially those associated with difficulties in memory and concentration, are not fully understood. There is evidence that restricting sleep duration, total sleep deprivation, and REM sleep deprivation inhibit the proliferation of nerve cells, i.e., neurogenesis [3]. In a study by Neylan et al. of veterans with PTSD and co-occurring insomnia, the severity of insomnia was found to be associated with the CA3/dentate hippocampal subfield [4].

The biochemical and molecular basis of insufficient sleep is still being investigated. One possible explanation is the dysregulation of the neurotrophic signaling pathway. Neurotrophins (NTs) are molecules found throughout the body. NTs are indispensable in the process of neuro- and gliogenesis. They include brain-derived neurotrophic factor (BDNF), and neurotrophin-3 (NT3) and 4 (NT4), which are responsible for the growth and repair of nerve cells. Glial-cell-derived neurotrophic factor (GDNF), which is involved in the formation of glial cells, also plays a similar role. Some authors also include GDNF in the category of NTs due to its role in the development of the nervous system [5].

BDNF is an NT that regulates synaptic plasticity, neuronal excitability, and nociception [6]. NT3 and NT4 are proteins with similar properties and functions to BDNF. NT3 activates a variety of receptor groups, primarily including tropomyosin-related kinase C (TrkC), but also neurotrophin receptor p75 (p75NTR), TrkB, and TrkA. NT4 activates p75NTR and TrkB similarly to BDNF, duplicating its functions [7].

NTs are also involved in the biochemistry of sleep. BDNF increases serotonergic signaling, inducing spontaneous awakenings. A correlation has been observed between BDNF expression and the need for slow-wave sleep [8]. Sasaki et al. showed that intracerebroventricular administration of NT3 and NT4 to rabbits resulted in an increase in the amount of time spent in the NREM sleep phase. NT4 also increased slow-wave activity [9]. Intracerebroventricular injection of GDNF extended the NREM sleep phase in rats and rabbits. A high dose of GDNF inhibits REM sleep in rabbits [10]. However, most studies on the role of NT in sleep are limited to basic research in animal models.

Previous studies, particularly in animal models, suggest a relationship between NT production and total sleep time. However, these studies are limited and often inconclusive. People who sleep less than 6 h had lower levels of BDNF compared to healthy controls [11]. However, acute sleep deprivation led to an increase in the concentration of BDNF and GDNF in the blood serum [12]. There are a lack of similar studies on NT3 and NT4. A correlation between serum BDNF levels and the severity of cognitive impairment in obstructive sleep apnea (OSA) has also been observed [13]. Reduced BDNF levels have also been shown in patients with Parkinson’s disease with restless legs syndrome (RLS) compared to those with Parkinson’s disease without RLS [14]. This NT was also elevated in narcolepsy. However, there are a lack of similar studies in the context of other NTs [15].

The next objective parameter assessed during polysomnography that can be linked to NTs is the strength of electroencephalographic waves in electroencephalography (EEG). EEG is a non-invasive measurement of the electrical fields of the brain. Electrodes placed on the head record the potential field from the electrical current in neurons and around them [16]. EEG is used in neurological diagnostics, but it can also be helpful in the differential diagnosis of sleep disorders such as insomnia, obstructive sleep apnea, narcolepsy, and parasomnias. Changes in EEG are observed in different sleep phases: alpha and theta waves dominate in the N1 NREM phase; theta waves, sleep spindles, and K complexes dominate in the N2 phase; delta waves dominate in the N3 phase; and low-voltage mixed-frequency EEG activity without sleep spindles and K complexes is observed in the REM phase [17].

EEG power represents the magnitude of activity in certain frequency bands of the signal, while coherence between different electrodes reflects the degree to which connections are present in brain areas [18]. One of the best-known indicators of sleep need is slow-wave activity, which is the power of EEG waves in the range of 0.5 to 4 Hz. In one study, after unilateral microinjections of BDNF into the cerebral cortex of rats, SWA during NREM sleep was higher in the injected hemisphere compared to the contralateral hemisphere [19]. Differences in EEG power have been observed between patients with insomnia and well-sleeping individuals [20]. However, subjective sleep quality does not seem to be associated with differences in EEG power [21]. In studies performed in the waking state, a relationship between EEG power and BDNF has been observed [22]. There are a lack of similar studies in the context of the remaining NTs, as well as those performed during sleep.

Therefore, the aim of this study was to investigate the relationship between PSG sleep parameters and EEG power spectral and selected NTs represented by protein concentration or gene expression.

## 2. Materials and Methods

The study was conducted at the Department of Sleep Medicine at the Medical University of Lodz, Poland, where all participants underwent polysomnography (PSG). Recruitment for the study was carried out through the scientific team’s social media profile and the snowball sampling method.

The Medical University of Lodz Bioethics Committee approved the study protocol (number: RNN/302/20/KE). All participants provided written informed consent.

A total of 113 participants were included in the study, of whom, 32 did not complete the entire research protocol.

To be included in the study, participants had to meet the following criteria: they had to sign an informed consent form; have a body mass index (BMI) of 20–30 kg/m^2^; and be between the age of 18 and 35 years. Participants were excluded from the study if they met any of the following criteria: pregnancy; breastfeeding; surgery in the last 6 months; diagnosis of an endocrine disease; a metabolic disorder; a chronic inflammatory disorder; a tumor (excluding basal-cell carcinoma); a history of radio/chemotherapy; renal, respiratory, or circulatory insufficiency; actively treated psychiatric disease; substance abuse; or an intercontinental flight during the examination or within 2 weeks before the screening of the study. In addition, patients were instructed to discontinue the use of hypnotic medications at least 14 days prior to undergoing PSG.

Individuals with prior diagnoses of narcolepsy, sleep apnea, restless legs syndrome, or insomnia within the past four weeks were excluded from the study. The presence of insomnia symptoms during the study screening process was evaluated using the Athens insomnia scale (the cut-off point was 6 or more points). 

Participants underwent a physical examination, including an assessment of body mass, height, and medical history. The PSG began around 10 p.m. and lasted until 7 a.m. (±15 min) the next day in the sleep center. The following routine PSG parameters were assessed: chin and limb electromyography (EMG), electroencephalography (EEG), electrooculography (EOG), electrocardiography (leads V1 and V2), body position, airflow signals (oronasal flow; thermistor gauge), oxygen saturation (SpO_2_), and respiratory effort signals from the chest and abdomen (piezoelectric gauges). Sleep latency was calculated as the time from lights out to the onset of NREM stage N1 sleep. Sleep efficiency was defined as the ratio of the duration of sleep time to the total time in bed (TIB).

PSG was performed using an Alice 4 device (Phillips Respironics, Murrysville, Pennsylvania, United States). Sleep stages were scored by the same experienced researcher according to the American Academy of Sleep Medicine (AASM) guidelines using a 30 s epoch standard in order to detect sleep-disordered breathing, arousals, periodic limb movements, or any other abnormalities [23]. EEG channels with an electrode impedance upper 5 KΩ were removed from the analysis. The data sampling rate was 200 Hz. Acquired signals were then exported to EDF files and edited, processed, and analyzed using the NeuroAnalyzer v0.23.9 (https://neuroanalyzer.org (accessed on 8 January 2024)) toolbox [24]. All channels have been referenced to the contralateral mastoid electrodes (e.g., O2A1 and O1A2). First, segments outside the detected REM periods were trimmed out. A total number of 311 REM segments were used for further analysis. Next, the pipeline included low-pass FIR filtering at 30 Hz and epoching into 20 s segments (resulting in a total of 2091 epochs from all EEG recordings). Finally, for each epoch, the power spectrum was calculated using the Welch periodogram (normalized to dB) and integrated using the composite Simpson’s rule at delta (0.1–4 Hz), theta (4–8 Hz), alpha (8–13 Hz), and beta (14–30 Hz) frequency ranges at F3, F4, C3, C4, O1, O2 locations (the position of the electrodes was in accordance with the international 10/20 system). Finally, for each subject, REM epoch band powers were averaged for each band and analyzed statistically. The EEG processing script is available in the https://codeberg.org/AdamWysokinski/research-data (accessed on 8 January 2024) repository.

Blood samples were collected from the peripheral circulation in the morning after the PSG examination. The samples were collected into tubes containing a clot activator and centrifuged immediately at 4 degrees Celsius. The serum was collected and stored at −80 °C. The serum BDNF, GDNF, NT3, and NT4 protein concentrations were measured using ELISA kits: Human BDNF ELISA Kit and Human GDNF ELISA Kit from FineTest, Wuhan, China; and Human NTF3 ELISA Kit and Human NTF4 ELISA Kit from EIAab Science Wuhan, China. The absorbance was measured at a wavelength of 450 nanometers using an absorbance reader (BioTek 800 TS, Agilent Technologies, Santa Clara, CA, USA).

RNA was isolated from the peripheral blood leukocytes (PBLs) using the TRIzol reagent (Invitrogen, Waltham, MA, USA). The RNA integrity number (RIN) and concentration of the isolated RNA were assessed using a Nanodrop Colibri Microvolume Spectrometer (Titertek Berthold, Pforzheim, Germany). The obtained material was reverse-transcribed using a dedicated kit (SuperScript IV First-Strand Synthesis System, Thermo Fisher Scientific Inc., Pleasanton, CA, USA) according to the manufacturer’s protocol. The reverse-transcription process consisted of three steps, each being carried out at 60 °C for 60 s. The level of expression of the chosen genes was determined by quantitative real-time polymerase chain reaction (qPCR). The qPCR mixture consisted of nuclease-free water, Fast TaqMan Universal Master Mix, cDNA, and gene-specific probes (TaqMan assays for BDNF, GDNF, NT3, NT4, and the reference gene β-Actin). The cycle threshold (CT) was calculated for each sample. The results were presented as ΔCT and analyzed using the Livak method [25].

Statistica 13.1 PL (StatSoft, Tulsa, OK, USA) was used to analyze the data. The significance level was set at *p* < 0.05. The distribution of continuous variables was assessed using the Shapiro–Wilk test. Data were presented as the median with interquartile range (IQR: first-third quartile) or mean with standard deviation (SD) for non-normal and normal distributions, respectively. Spearman’s correlation test was used to assess correlations. Parametric independent variables were assessed using the *t*-test. The Mann–Whitney U test or Wilcoxon signed-rank test was used for nonparametric independent or dependent variables, respectively.

## 3. Results

There were 81 participants included in the analysis. Demographic data of the participants are presented in Table 1. A nearly equal number of men and women were enrolled in the study. The median age was 24 years. The median total sleep time was 407 min, while the median sleep latency and sleep efficiency were 35 min and 78.6%, respectively. PSG analysis revealed no instances of sleep-disordered breathing, arousals, periodic limb movements, or other abnormalities meeting the AASM criteria among the participants included in the analysis. 

A correlation analysis of neurotrophic factors with PSG parameters showed a negative correlation between TIB and BDNF protein levels (r = −0.37, *p* = 0.001), but a positive correlation with *BDNF* mRNA (r = 0.24, *p* = 0.041). In addition, NT4 and GDNF proteins negatively correlated with TIB (r = −0.32, *p* = 0.005; r = −0.25, *p* = 0.032, respectively), but not with mRNA. The remaining neurotrophic factors did not correlate with TIB (Table 2).

Total sleep time, duration of NREM and REM sleep, sleep efficiency, and REM sleep latency did not correlate with the studied parameters. However, sleep latency negatively correlated only with *GDNF* mRNA (r = −0.25, *p* = 0.035). Non-REM events negatively correlated with BDNF and NT3 proteins, but not with mRNA expression (r = −0.24, *p* = 0.037; r = −0.24, *p* = 0.037, respectively). REM events correlated positively only with NT4 protein levels (r = 0.32, *p* = 0.005; Table 2).

EEG recordings that did not meet the quality criteria were excluded from the analysis. A summary of the analyzed data is presented in Table 3. The analysis of EEG power with selected neurotrophic factors showed that only the BNDF protein is associated with EEG power, but not other neurotrophins or *BDNF* mRNA. Table 3 shows that the BDNF protein level correlates negatively with delta power waves in all studied leads during REM sleep. During NREM sleep, a negative correlation with delta power waves was observed in leads located over the left hemisphere of the brain (F3, C3).

## 4. Discussion

Current knowledge about the effects of sleep and its quality on neurotrophic processes is still very limited. There is a particular lack of research that takes into account objective measures of sleep, such as brain bioelectrical activity, which varies depending on the sleep stage [26]. 

In our study, we found that the levels of the proteins BDNF, NT4, and GDNF, but not NT3, may be affected by TIB. Only BDNF mRNA expression was affected by TIB. None of the neurotrophins correlated with the duration of efficient sleep, REM and NREM sleep. 

BDNF protein levels negatively correlated with TIB, while BDNF mRNA levels positively correlated with this parameter. The number of NREM sleep disruption episodes also affected BDNF protein levels. As mentioned above, one study found a correlation between BDNF and sleep time, but the study participants were patients with insomnia symptoms. It has been shown that it is not insomnia itself, but a short sleep duration that reduces BDNF levels [11]. 

A different study found a negative correlation between BDNF and the severity of insomnia symptoms, as measured by the Insomnia Severity Index [27]. However, this preliminary study did not examine which specific insomnia symptoms specifically affect BDNF levels. The above relationships suggest that sleep deprivation increases BDNF transcription processes while also using BDNF proteins. Similar studies are lacking, especially those that take into account objective sleep parameters. Additionally, to our knowledge, this is the first study to evaluate the relationship between BDNF mRNA expression and insomnia symptoms.

The literature on the remaining neurotrophins is even more limited. For example, we found a negative correlation between GDNF and TIB. Previous studies have shown that GDNF promotes sleep, especially NREM, but there is no evidence of a link between GDNF and insomnia symptoms [10]. GDNF plays a similar role in the body as BDNF, regulating the dopaminergic system, but its highest concentrations in the body are different from those of BDNF [28]. The above results suggest that GDNF, like BDNF, is also associated with TIB, but not with the sleep duration.

Unlike BDNF and NT3, NT4 protein levels were negatively correlated with REM sleep disruption episodes, but not NREM sleep disruption episodes. These results are based on the knowledge of how these molecules interact with each other. BDNF needs other neurotrophins, including NT3, to function biologically. The differences between BDNF and NTF4 in the context of sleep events may be explained by the fact that, although both molecules bind to and activate TrkB receptors, they mediate different neuronal functions. The molecular mechanism of how TrkB activation by BDNF and NT4 leads to different outcomes is unknown. It is likely that BDNF and NT4 lead to the different sorting of TrkB receptors within cells, which results in different biological functions in cultured cortical neurons [29].

We also found that lower BDNF protein levels were associated with NREM episodes. This is supported by other studies, which have shown that BDNF plays a role in regulating NREM sleep [19,30]. For example, one study found that increased endogenous BDNF levels were associated with increased slow-wave activity during recovery NREM sleep after total sleep deprivation [19]. However, other studies have found that lower BDNF levels are associated with greater REM sleep events. In BDNF heterozygous rats, REM sleep episodes were shorter and took longer to initiate than in wild-type rats. The results of our study suggest that BDNF may play a role in the regulation of the quantity of both NREM and REM events. However, further studies are needed to confirm this association and to elucidate the underlying mechanisms [31].

The above results seem to be confirmed by our EEG power analysis with BDNF concentration and expression. We observed that BDNF is negatively correlated with the EEG power of delta waves in REM sleep. This is similar in NREM sleep, but only from the left hemisphere. There have been a lack of similar studies in the context of sleep. In the waking state, in one study, Roy et al. observed a focal increase in the right frontotemporal delta and a decrease in the amplitude of alpha waves in the BDNF Met/Met genotype group compared to the Val/Val and Val/Met groups, which are particularly linked to neuroplasticity and cognitive function. Stronger frontal topographies were shown for beta waves in the Val/Met group compared to the Val/Val group [22]. It is also known that BDNF can affect REM sleep homeostasis [32].

The observed differences can also be explained by the model of transcranial direct current stimulation (tDCS). Besides the direct effect of tDCS on the resting neuronal potential, the long-term aftereffects of tDCS result mostly from stimulating various neurotrophic mechanisms [33] (although tDCS does not seem to directly increase BDNF levels, but rather its effectiveness depends on the baseline BDNF level [24,34]). In many previous studies, delta power consistently decreased after tDCS treatment, e.g., in [35,36,37]. Therefore, it seems that the observed negative correlation with the delta band power may be due to the fact that increased delta power is associated with decreased efficiency of the neurotrophic mechanisms.

Our knowledge of the remaining neurotrophins is very limited. In one study, GDNF supplementation reduced epileptic foci in the EEG recording [38]. Only in a rat model was it observed that the administration of NT3 reduced the power of EEG waves, mainly delta and beta, and also increased the amount of NREM sleep [39]. In our study, we did not observe a correlation between NT3, NT4, and GDNF proteins and EEG power. It seems that, despite the fact that BDNF has similar properties and functions to NT3 and NT4, BDNF plays a more important role in regulating the power of EEG waves.

The present study has several limitations. On the one hand, the study of neurotrophins in the peripheral blood may be subject to error; however, it is known that neurotrophins pass through the blood–brain barrier and their serum concentrations are correlated. Measurement of neurotrophin concentrations in the peripheral blood allowed for the recruitment of a larger number of healthy volunteers. Additionally, several-hour EEG recordings during sleep are at risk of a loss of recording quality, and therefore of drawing incorrect conclusions; we tried to reduce this limitation by pre-analyzing individual EEG channels for their quality by removing channels with higher impedance. A limitation of the study is also that only one PSG study was conducted on each participant under laboratory conditions, which could have significantly affected parameters such as sleep latency or the relatively low sleep efficiency obtained in this study. Additionally, please note that the delta range in our analysis was defined as 0.1–4 Hz, while it is sometimes defined as 0.5–4 Hz. The inclusion of the 0.1–0.4 Hz range might increase the total power in the delta range, yet it is doubtful that it affects the general conclusions.

The presented results indicate that the concentrations and expression of neurotrophins are dependent on TIB and sleep events (such as awakening, hypopnea, movements), but not their quantity. Increased BDNF and NT3 are associated with the amount of NREM events, while elevated NT4 is possibly positively associated with REM sleep; although, this issue requires further, more detailed research. BDNF is mainly associated with the power of delta waves in EEG, and its negative correlation with the power of these waves suggests that delta sleep is associated with a decrease in neurotrophic processes. The positive effect of sleep on cognitive processes and mood may have a neurotrophic basis associated with delta waves, which requires further research.

## Figures and Tables

**Table 1:** Baseline characteristics of study participants.

Women (n, %) Men (n, %)	41 (50.62) 40 (49.38)
Age	24.00 (22.00–26.00)
BMI	22.77 (21.47–24.76)
Smoking (n, %)	9 (11.11)
Higher education (n, %)	39 (48.15)
TIB (min)	536.00 (515.00–558.80)
TST (min)	407.00 (360.00–470.50)
REM duration (min)	91.00 (67.50–121.50)
NREM duration (min)	332.50 (285.50–352.50)
Sleep latency (min)	35.00 (22.00–59.00)
Latencies REM (min)	142.00 (94.50–185.50)
Sleep efficiency (%)	78.60 (70.20–86.20)
NREM events	3.00 (1.00–7.00)
REM events	3.00 (1.00–6.00)

Abbreviations: BMI: body mass index, NREM: non-rapid eye movement sleep, REM: rapid eye movement sleep, TIB: total time in bed, TST: total sleep time, min: minutes.

**Table 2:** Correlations between selected neurotrophic factors and polysomnographic parameters.

	TIB	TST	NREM Duration	REM Duration	Sleep Latency	REM Latencies	Sleep Efficiency	Non-REM Events	REM Events
BDNF	−0.37; 0.001	−0.21; 0.066	−0.15; 0.203	−0.19; 0.096	0.17; 0.131	0.06; 0.593	−0.02; 0.837	−0.24; 0.037	−0.15; 0.206
mRNA BDNF	0.24; 0.041	0.04; 0.709	0.05; 0.675	0.01; 0.945	−0.14; 0.225	−0.13; 0.273	−0.05; 0.684	−0.05; 0.673	−0.18; 0.141
NT3	0.05; 0.689	−0.2; 0.085	−0.17; 0.146	−0.16; 0.157	0.01; 0.914	0.17; 0.149	−0.01; 0.91	−0.24; 0.041	−0.09; 0.449
mRNA NT3	0.11; 0.357	0.1; 0.416	0.13; 0.286	−0.04; 0.748	−0.2; 0.091	0; 0.985	0.05; 0.679	−0.19; 0.11	−0.22; 0.062
NT4	−0.32; 0.005	−0.05; 0.659	−0.15; 0.191	0.11; 0.325	0.04; 0.762	−0.13; 0.247	−0.1; 0.392	0.16; 0.162	0.32; 0.005
mRNA NT4	0.01; 0.901	0.07; 0.563	0.09; 0.469	−0.04; 0.745	−0.07; 0.556	−0.07; 0.569	0.05; 0.695	−0.03; 0.776	−0.2; 0.095
GDNF	−0.25; 0.032	−0.17; 0.141	−0.18; 0.119	0.01; 0.943	−0.08; 0.517	−0.2; 0.091	−0.07; 0.572	−0.1; 0.369	−0.18; 0.13
mRNA GDNF	0.09; 0.431	0.04; 0.719	0.07; 0.587	−0.05; 0.675	−0.25; 0.035	0.07; 0.539	0; 0.978	−0.15; 0.204	−0.17; 0.162

The data are presented as: r; p. Abbreviations: BDNF: brain-derived neurotrophic factor, GDNF: glial-cell-derived neurotrophic factor, NREM: non-rapid eye movement sleep, mRNA: messenger RNA, NT3: neurotrophin-3, NT4: neurotrophin 4, REM: rapid eye movement sleep, TIB: total time in bed, TST: total sleep time.

**Table 3:** Correlations between selected neurotrophic factors and the power of electroencephalographic waves recorded during polysomnography.

	n	BDNF	mRNA BDNF	NT3	mRNA NT3	NT4	mRNA NT4	GDNF	mRNA GDNF
REM delta F4A1	43	−0.48; 0.001	0.09; 0.590	0.05; 0.74	0.02; 0.896	−0.19; 0.238	0.07; 0.69	0.09; 0.567	−0.04; 0.813
REM delta F3A2	53	−0.37; 0.008	0.15; 0.312	0.07; 0.637	0.03; 0.855	−0.11; 0.43	0.14; 0.338	0.18; 0.205	−0.02; 0.867
REM delta C4A1	43	−0.43; 0.005	0.14; 0.392	0.04; 0.781	0.03; 0.85	−0.11; 0.476	0; 0.978	0.11; 0.494	0.02; 0.917
REM delta C3A2	53	−0.36; 0.01	0.15; 0.317	0.06; 0.666	0.03; 0.822	−0.11; 0.424	0.14; 0.342	0.19; 0.184	−0.02; 0.912
REM delta O2A1	40	−0.38; 0.017	0.09; 0.582	−0.02; 0.923	0.03; 0.848	−0.11; 0.506	0.05; 0.785	0.17; 0.298	0; 0.991
REM delta O1A2	46	−0.36; 0.014	0.12; 0.450	0.21; 0.169	−0.04; 0.785	0.01; 0.923	−0.12; 0.436	0.14; 0.363	−0.04; 0.779
REM theta F4A1	41	−0.01; 0.943	0.07; 0.683	−0.08; 0.607	0.14; 0.392	0.19; 0.236	−0.03; 0.843	0.1; 0.521	0.18; 0.277
REM theta F3A2	49	0.09; 0.543	−0.03; 0.823	−0.03; 0.829	−0.15; 0.323	0.11; 0.44	−0.09; 0.535	0.17; 0.239	−0.04; 0.781
REM theta C4A1	39	0.04; 0.791	0.05; 0.790	−0.08; 0.633	0.16; 0.366	0.25; 0.135	−0.05; 0.778	0.08; 0.625	0.19; 0.272
REM theta C3A2	49	0.04; 0.773	−0.03; 0.867	−0.08; 0.602	−0.11; 0.488	0.09; 0.563	−0.08; 0.609	0.15; 0.325	−0.04; 0.807
REM theta O2A1	37	−0.14; 0.414	0.21; 0.226	0.1; 0.55	0.29; 0.1	−0.04; 0.8	0.12; 0.495	−0.01; 0.944	0.26; 0.138
REM theta O1A2	39	−0.28; 0.089	0.02; 0.886	0.15; 0.384	−0.04; 0.805	−0.11; 0.498	−0.1; 0.553	0.09; 0.59	−0.01; 0.97
REM alpha F4A1	39	−0.11; 0.492	0.29; 0.081	−0.1; 0.544	0.12; 0.49	0.01; 0.967	−0.01; 0.972	0.01; 0.955	0.19; 0.25
REM alpha F3A2	50	0; 0.987	0.03; 0.853	−0.01; 0.958	−0.12; 0.425	−0.01; 0.954	−0.04; 0.797	0.21; 0.143	−0.08; 0.577
REM alpha C4A1	41	−0.06; 0.715	0.2; 0.240	−0.18; 0.258	0.09; 0.577	0.06; 0.696	−0.07; 0.656	0.11; 0.498	0.14; 0.399
REM alpha C3A2	38	−0.02; 0.897	0.04; 0.788	−0.07; 0.643	−0.16; 0.284	−0.02; 0.892	−0.11; 0.483	0.18; 0.221	−0.12; 0.452
REM alpha O2A1	39	−0.03; 0.855	0.14; 0.425	−0.02; 0.91	0.23; 0.182	0.09; 0.608	0.11; 0.545	0.1; 0.568	0.2; 0.251
REM alpha O1A2	49	−0.09; 0.58	−0.07; 0.661	0.12; 0.463	−0.09; 0.61	0.01; 0.961	−0.1; 0.54	0.18; 0.27	−0.09; 0.586
REM beta F4A1	44	−0.22; 0.152	0.11; 0.487	−0.01; 0.967	0.07; 0.682	−0.1; 0.538	0.1; 0.527	0.27; 0.085	0.13; 0.41
REM beta F3A2	51	−0.09; 0.516	−0.14; 0.333	0.07; 0.65	−0.16; 0.275	0.01; 0.949	0.01; 0.96	0.24; 0.097	−0.14; 0.34
REM beta C3A2	44	−0.08; 0.568	0.11; 0.505	0.02; 0.914	0.08; 0.629	0.02; 0.875	0.08; 0.615	0.24; 0.087	0.17; 0.284
REM beta C4A1	51	−0.18; 0.235	−0.1; 0.488	−0.05; 0.739	−0.1; 0.517	−0.09; 0.567	0.09; 0.546	0.3; 0.051	−0.08; 0.581
REM beta O2A1	40	−0.19; 0.255	0.03; 0.868	−0.04; 0.825	0; 0.986	−0.07; 0.668	0.02; 0.906	0.25; 0.118	0; 0.98
REM beta O1A2	44	−0.28; 0.065	−0.02; 0.895	0.07; 0.661	0.07; 0.675	−0.13; 0.407	−0.01; 0.972	0.2; 0.194	0.09; 0.591
NREM delta F4A1	44	−0.23; 0.134	0.19; 0.226	−0.13; 0.401	0.13; 0.419	−0.04; 0.806	0.17; 0.285	0.13; 0.4	0.03; 0.863
NREM delta F3A2	52	−0.42; 0.002	0.22; 0.127	−0.18; 0.196	0; 0.989	−0.2; 0.164	0.11; 0.452	0.04; 0.769	−0.12; 0.433
NREM delta C3A2	52	−0.42; 0.002	0.22; 0.124	−0.18; 0.214	0; 0.98	−0.2; 0.158	0.11; 0.444	0.06; 0.698	−0.11; 0.44
NREM delta C4A1	44	−0.18; 0.236	0.26; 0.099	−0.11; 0.491	0.15; 0.341	0.02; 0.897	0.15; 0.35	0.18; 0.255	0.09; 0.563
NREM delta O2A1	40	0.08; 0.64	−0.13; 0.431	−0.01; 0.934	−0.06; 0.742	−0.08; 0.62	−0.18; 0.273	0.19; 0.241	0.02; 0.886
NREM delta O1A2	46	−0.26; 0.09	0.10; 0.532	0.16; 0.299	−0.03; 0.83	−0.12; 0.444	−0.18; 0.263	0; 0.995	−0.03; 0.829
NREM theta F4A1	44	0.1; 0.533	0.12; 0.460	−0.11; 0.474	0.05; 0.755	0.18; 0.25	−0.07; 0.663	0.05; 0.73	0.09; 0.563
NREM theta F3A2	52	−0.1; 0.489	−0.02; 0.867	−0.04; 0.755	−0.09; 0.552	−0.08; 0.583	−0.03; 0.826	0.09; 0.543	−0.11; 0.459
NREM theta C4A1	44	0.06; 0.683	0.10; 0.531	−0.16; 0.308	0.04; 0.781	0.21; 0.179	−0.05; 0.737	0.05; 0.733	0.07; 0.679
NREM theta C3A2	52	−0.15; 0.306	−0.01; 0.930	−0.09; 0.547	−0.07; 0.627	−0.07; 0.616	0.04; 0.763	0.12; 0.389	−0.1; 0.483
NREM theta O2A1	40	0.25; 0.132	−0.28; 0.091	0.05; 0.754	−0.15; 0.385	0.07; 0.669	−0.25; 0.136	0.18; 0.271	−0.11; 0.536
NREM theta O1A2	45	−0.06; 0.676	−0.01; 0.925	−0.07; 0.655	−0.01; 0.947	−0.05; 0.735	−0.12; 0.44	−0.09; 0.546	0; 0.986
NREM alpha F4A1	44	0.17; 0.283	0.14; 0.390	−0.12; 0.456	0.11; 0.478	0.12; 0.444	−0.06; 0.732	0.04; 0.817	0.16; 0.308
NREM alpha F3A2	51	0.1; 0.488	0.10; 0.515	−0.04; 0.758	0.01; 0.942	0.1; 0.503	0; 0.982	0.01; 0.942	0; 0.999
NREM alpha C4A1	44	0.13; 0.424	0.16; 0.303	−0.2; 0.208	0.13; 0.407	0.17; 0.266	−0.01; 0.935	0.04; 0.809	0.17; 0.28
NREM alpha C3A2	51	0; 0.976	0.13; 0.373	−0.13; 0.366	0.03; 0.831	0.08; 0.574	0.09; 0.564	0.1; 0.474	0.02; 0.891
NREM alpha O2A1	40	0.18; 0.277	−0.16; 0.338	0.01; 0.942	−0.01; 0.949	−0.02; 0.882	−0.2; 0.238	0.16; 0.325	0.04; 0.794
NREM alpha O1A2	45	−0.01; 0.96	−0.01; 0.974	−0.02; 0.912	−0.01; 0.97	−0.06; 0.682	−0.07; 0.642	−0.04; 0.806	0.01; 0.969
NREM beta F4A1	43	0.07; 0.649	0.10; 0.516	0.03; 0.85	0.16; 0.323	−0.12; 0.46	−0.05; 0.779	0.13; 0.428	0.25; 0.11
NREM beta F3A2	51	0.26; 0.068	−0.12; 0.438	0.1; 0.495	0.02; 0.881	0.11; 0.459	−0.14; 0.345	0; 0.999	0.05; 0.748
NREM beta C4A1	44	0.04; 0.794	0.12; 0.447	−0.13; 0.404	0.15; 0.346	−0.04; 0.815	0.01; 0.929	0.28; 0.068	0.29; 0.068
NREM beta C3A2	51	0.12; 0.407	−0.09; 0.554	−0.02; 0.911	0.08; 0.61	0.13; 0.366	−0.01; 0.944	0.08; 0.599	0.06; 0.667
NREM beta O2A1	40	0; 0.999	−0.15; 0.372	−0.03; 0.849	0.08; 0.64	−0.21; 0.207	−0.13; 0.453	0.31; 0.055	0.17; 0.321
NREM beta O1A2	45	−0.09; 0.574	0.04; 0.824	−0.04; 0.8	0.05; 0.762	−0.16; 0.311	−0.03; 0.873	0.06; 0.688	0.06; 0.69

The data are presented as: r; p. BDNF: brain-derived neurotrophic factor, GDNF: glial-cell-derived neurotrophic factor, NREM: non-rapid eye movement sleep, mRNA: messenger RNA, NT3: neurotrophin-3, NT4: neurotrophin-4, REM: rapid eye movement sleep. F4A1, F3A2, C4A1, C3A2, O2A1, and O1A2 represent EEG channels.

## Data Availability

Data are available on request from the authors due to privacy restrictions.
